# Collaboration with the Laboratory Automation Standards Foundation

**DOI:** 10.1155/S1463924695000290

**Published:** 1995


Journal of Automatic Chemistry, Vol. 17, No. 5 (September–October 1995), pp. 191–193

### Collaboration with the Laboratory Automation Standards Foundation

***Editorial***
***A partnership for the development of automation strategies***

**Professor P. B. Stockwell**
*P S Analytical Ltd, Arthur House, Unit 3 Crayfields Industrial Estate, Main Road, Orpington, Kent BR5 3HP, UK*

This issue of the journal sees the first of what I hope will be many contributions from LASF—the Laboratory Automation and Standards Foundation. The LASF is one of many groups working towards the goal which readers of the journal share with me. People are approaching the ideal in a number of ways and it seems to me that one of the aims of the journal should be to share experiences and provide pieces of the jigsaw.

I began with automation in a working laboratory, looking at problems of sampling and sample preparation but with limited resources available in terms of computing and software. The current situation with respect to ease of use and availability of computers and software was recently compared with the situation in the 1970s and 1980s [1].

I was part of a group (at the UK's Laboratory of the Government Chemist) designing and developing automatic systems. The group's philosophy was based on our own understanding and backgrounds but this philosophy still offers significant information and advantages for other uses. We tried to produce 'good automation', getting the level of sophistication right. We tried not to over-complicate matters and evolved a 'Total Systems Concept' [2], which set out to involve all aspects of the analytical process from sample collection, pre-treatment measurement and instrument control through to the calculation, validation and reporting required for the methods. Constraints imposed by organizations and the political sensitivity of any situation would obviously be included in the manner in which the analyses could be automated. Of course statutory requirements have to be met, particularly for test case issues, but screening by an alternative, more readily automated method, may well be a possibility. Clearly an automation project requires a multi-disciplinary approach, but above all the analyst will play a crucial role, the analyst alone being able to specify the constraints imposed by the chemical regime. Team co-operation is vital and engineering principles must be included in this team approach. The final automated instrument must not be a Heath Robinson design: it must be easy to use, simple to maintain and friendly for the user. Indeed, it is often vital for the user to be fully committed to the introduction of the instrumentation before the programme is begun. These aims are highlighted in table 1.

*Table 1. Aim of automation group.*

| | | |
|---|---|---|
| (a) | Good automation | At correct level of sophistication used |
| (b) | Total system approach | From sample preparation through to reporting |
| (c) | Engineered version | Simple to operate |

The interactions between the various participants of an automation team and a client group have been outlined previously [3]. Discussing these issues with Joe Liscouski of LASF (see below) has shown how very similar his views are to my own. I hope that the co-operation will last for a significant period and that each group will gain greatly from this. With more emphasis on instrument and software validation, it becomes even more important that specialists across the globe should be working towards acceptable and common goals.

### References

1. Stockwell, P. B., *Journal of Automatic Chemistry*, **16** (1994), 155.

2. Stockwell, P. B., *Journal of Automatic Chemistry*, **1** (1979), 216.

3. Stockwell, P. B., *Journal of Automatic Chemistry*, **1** (1978), 10.

***Engineering laboratory automation***

**Joe Liscouski**
*Laboratory Automation Standards Foundation, PO Box 38, Groton, MA 01450, USA*

We need to change the way we approach laboratory automation and computing. Technologies that were once the research tools of a few daring souls, are now commonplace and used by many. Our acceptance of these tools has improved the way we work, but there is clear evidence that customers are not getting the benefits they expected: projects are taking too long to implement, they are becoming too expensive, integration between systems is difficult, the failure rate of first attempts in automation is high and people's expectations of what can

0142-0453/95 $10.00 © 1995 Taylor & Francis Ltd.

be done are often unrealistic. I have recently encountered a number of problems, for example a robotics project that was expected to take a few weeks (an almost 'turnkey' system), but took over eight months to complete; people trying to implement complex databases using spreadsheets because that was all they knew; people on their third LIMS implementations because their initial plans did not work out; and sales representatives who did not understand the basic principles on which their products function. Unfortunately, this is not new or unusual.

One of the first things that needs to be recognized is that laboratory automation is not a part-time job. Laboratory automation is not a science, it is an *engineering discipline* in support of, and in pursuit of, science. If people are going to do work in this field, they need to be trained as engineers. That means learning how to design and build systems, how to test them and demonstrate that they work before they are put into use. It also means learning the capabilities of the tools available, how to apply them and how to develop new tools when needed. Business managers need to learn how to manage strategic and tactical uses of automated systems and how to create the infrastructure necessary to make projects successful. In short, we need to develop a separate technical discipline of *laboratory automation engineering*, which covers systems design, information technologies, computing, networks, databases, robotics, graphics, human factors management and the sciences that engineering work is being applied to. If implemented as a degree-level programme, it should be at the graduate level only, built on a base of computer science and chemistry, biology, physics, or another engineering discipline. You have to have a thorough understanding of what an engineering activity is being applied to before you apply it. If an engineering mind-set were applied to laboratory automation problems, most of the issues over validation would evaporate; 'validation' is a normal part of a proper system's engineering.

A second thrust is to further burden undergraduate programmes in science by requiring a period of laboratory computing as a prerequisite for obtaining a degree. Computer-assisted laboratory instrumentation is now found in every laboratory. How many students understand the devices that are governing their ability to acquire, analyse and manage data? Do they know what an A/D is, what kinds of A/Ds are available, which ones are best applied to what problems and how wiring options can affect the quality of their data? Do they know what a LIMS is, what its role in a laboratory is, the kinds of choices of systems? Do they understand the role of robotics? In short, are they being properly prepared to work effectively in today's academic and industrial sciences?

The marketing thrust of many computer and software vendors is to treat systems as appliances—just plug them in, turn them on, and enjoy. However, there are huge problems, for example the Pentium fiasco and people running into software conflicts between different products.

These systems are not appliances and attempts to portray them as such for all but the simplest uses are inappropriate. For laboratory work it may result in the disqualification of data. Scientists need to approach laboratory computing with scepticism. Use it once you are satisfied that it works. Take the training necessary to understand what questions to ask and to evaluate the answers. That training should be part of every scientist's education.

Part of the work of the Laboratory Automation Standards Foundation (LASF) is to help educate people in the use and application of automation technologies. We are in the process of examining the training needed for an effective undergraduate laboratory automation course and for a broader set of courses for laboratory automation professionals. We invite your suggestions and opinions on these issues. Please contact us at LASF, PO Box 38, Groton, MA 01450, USA; EMAIL: joel66@aol.com; tel.: (USA) 508 448 6130.

---

### *3rd Annual Conference on the Validation of Laboratory Systems: October 1995*

The Laboratory Standards Foundation's third conference on validation will look at designing and implementing an effective laboratory validation programme:

- How do you design an effective process validation and equipment qualification programme?
- When do you begin?
- How have others approached the problem?
- What can vendors do to help?
- What do regulatory agencies expect?
- What changes may take place in requirements?
- How will technology changes affect the work?

Participants will include regulatory officials, end-users, system vendors and organizations that can help (for example LASF and ASTM).

The meeting will be held from 10 to 12 October 1995 in New Hampshire, and will cover vendor audits, validation of data acquisition systems; and data interchange standards.

### *Conducting a vendor audit*

The FDA is considering the addition of a 'vendor audit' requirement to GLPs and GMPs. This would increase the cost of purchasing and installing computer hardware, software, and instrumentation. This session will look at the elements that go into a successful vendor audit.

Beginning with presentations on current hardware and software engineering practices as a foundation, vendors and users will describe their views on the conduct of an audit. The impact of ISO 9000 certification will then be considered and there will be an opportunity to hear what a registrar does when a company is audited for ISO 9000 certification. These presentations will provide the basis for an open discussion on any aspect of the process, including the options available for independent third-party evaluations of vendors.

The FDA is concerned with the impact that requiring the institution of a vendor audit programme will have on both the vendor and end-user community. Using third parties to provide an independent audit programme will considerably simplify the process and reduce the overall cost and impact. The elements that an independent vendor evaluation programme should have will be considered as part of the discussion.

*Validation of data acquisition systems*

Data acquisition systems come in two forms: ready-to-use as provided by the vendor, and kits (individual choices of hardware, software, programming). This session will look at the methods that can be used to validate these systems.

*The impact of data format/interchange standards on raw data management*

FDA regulations are clear on the handling of raw data. Since they were drafted, laboratory work has changed and now includes scanned photographic images and the use of industry standard data interchange formats. It is not clear how industry standard formats for holding and exchanging data (for example TIFF, PICT, PCX, and EPS) can be used in FDA regulated environments since they require a change in the file format used to contain the data (the data elements themselves are either not changed, or are recast according to accepted and documented procedures).

The use of industry standard file formats—rather than the proprietary formats used as the primary data structures in most data acquisition systems—will permit the introduction of new software for data analysis and management. In addition, end-users will have more flexibility in the choice of analytical instrument and data system vendors. The use of standardized data formats will also benefit the regulatory agencies as well, for example, an investigator can use the same software to examine chromatographic data regardless of its origin. The uncertainty in the acceptance of data in standardized formats by regulatory agencies is hampering their widespread use, and in turn, their further development; companies are not going to commit data to a format that is not acceptable to an investigator.

The purpose of this session is to review the need for, and the benefits, of data interchange/format standards, and then, to explore what is needed to make them acceptable to regulatory agencies as primary raw data holders. This session could have significant impact on future developments in structuring laboratory automation systems.

The Laboratory Automation Standards Foundation, a non-profit organization, can be contacted at PO Box 38, Groton, Massachusetts 01450, tel: (508) 448 6130, or EMAIL [joel66@aol.com].

